# *In vitro* bioactivity and phytochemical characterization of a polyherbal extract with antioxidant and anticancer properties

**DOI:** 10.7717/peerj.20824

**Published:** 2026-02-17

**Authors:** Aiman Abdullah Ammari, Hossam Mohammed Aljawdah, Ramzi Ahmed Amran, Ahmad Rashed Alhimaidi

**Affiliations:** Department of Zoology, King Saud University College of Science, Riyadh, Saudi Arabia

**Keywords:** Sidr (*Ziziphus spina-christi*), Fenugreek (*Trigonella foenum-graecum*), Black Seed (*Nigella sativa*), Antioxidant activity, Phenolic compounds, Cytotoxicity

## Abstract

Herbal extracts are increasingly recognized for their therapeutic potential due to their rich phytochemical composition and associated bioactivities. This study evaluates the antioxidant and anticancer properties of a compound extract derived from Sidr (*Ziziphus spina-christi*), Fenugreek (*Trigonella foenum-graecum*), and Black Seed (*Nigella sativa*), Quantitative phytochemical analysis revealed high total phenolic (89.35 ± 0.36 mg GAE/g) and tannin content (88.60 ± 0.51 mg GAE/g), along with moderate flavonoid (28.67 ± 0.09 mg QE/g) and flavonol (10.52 ± 0.51 mg QE/g) levels, indicative of robust antioxidant potential. The extract demonstrated notable free-radical scavenging activity, with IC_5__0_ values of 359.93 ± 9.91 µg/mL and 292.93 ± 4.24 µg/mL in the 2,2-diphenyl-1-picrylhydrazyl (DPPH) and 2,2-azino-bis-3-ethylbenzothiazoline-6-sulfonic acid (ABTS) assays, respectively. Cytotoxicity evaluation against Caco-2 colorectal cancer cells showed an IC_5__0_ value of 103.40 ± 4.51 µg/mL, supporting its anticancer potential. UV–Vis spectral analysis identified a prominent peak at 323.14 nm, suggesting the presence of phenolic and aromatic compounds, while zeta potential analysis (−22.1 ± 6.92 mV) indicated moderate dispersion stability. These results provide preliminary evidence supporting the extract’s potential as a multifunctional bioactive formulation and offer a foundation for future mechanistic and *in vivo* investigations.

## Introduction

Natural products have long played a foundational role in drug discovery due to their structural diversity, biological specificity, and pharmacological potential. In recent years, interest in traditional medicinal plants has intensified owing to their demonstrated antioxidant, antimicrobial, and anticancer activities (Chaachouay & Zidane, 2024; Chunarkar-Patil et al., 2024). Marine- and land-derived botanicals continue to serve as a pivotal source of novel therapeutic compounds, supporting ongoing natural-product–based pharmaceutical development (Banday et al., 2024). Foundational literature further highlights the historical contribution of natural compounds to modern drug discovery platforms (Cragg & Newman, 2013; Thomford et al., 2018). Within this context, the combination of herbal extract represents a promising polyherbal approach, as each plant contains bioactive compounds with clinically relevant properties. Sidr, a member of the Rhamnaceae family, has been traditionally used for dermatological, inflammatory, and gastrointestinal illnesses. Its phytochemical profile includes flavonoids, alkaloids, saponins, and tannins, contributing to strong antioxidant and antimicrobial activity (Egigu & Mogesse, 2024; Abdel-Sattar et al., 2024). Several studies have also demonstrated anticancer effects associated with *Ziziphus* species, attributed to phenolic and alkaloid-mediated apoptosis pathways (Mongalo, Mashele & Makhafola, 2020; Pourahmadi et al., 2023). Fenugreek (*Trigonella foenum-graecum*), a widely cultivated Fabaceae herb, contains diverse bioactive constituents such as diosgenin and steroidal saponins, which contribute to anticancer, and hypoglycemic effects (Khatoon et al., 2024; Kalantari et al., 2024). Its antioxidant properties are particularly notable, reducing oxidative stress linked to metabolic disorders and tumorigenesis (Shadab, Akhtar & Siddiqui, 2024). Additional studies support its roles in lipid regulation and metabolic stability (Singh, Chaurasia & Bharati, 2023; Kumar et al., 2021).

Recent *in vivo* studies have demonstrated that fenugreek extracts exert sex-specific pharmacological effects, particularly in hepatic and renal tissues. Ammari et al. (2025a) reported dose- and sex-dependent modulation of liver enzymes and histological profiles in healthy rats, indicating metabolically active bio-components that influence hepatic function. Similarly, Ammari et al. (2025b) showed nephroprotective effects of fenugreek extract, with treatment improving kidney function markers in a sex-specific manner. These findings support the therapeutic relevance of fenugreek and provide biological context for examining its contribution within combined herbal formulations. Black Seed (*Nigella sativa*), known historically within Unani and Ayurvedic medical systems, contains thymoquinone, a key compound with well-documented antioxidant, anticancer effects (Sadeghi, Imenshahidi & Hosseinzadeh, 2023; Khalid et al., 2024). Other constituents, including flavonoids and essential oils, contribute to immune modulation and metabolic protection (Oubannin et al., 2024). Multiple studies have demonstrated its ability to inhibit cancer cell proliferation *via* oxidative stress modulation, apoptosis induction, and signal transduction interference (Sheikhnia et al., 2023; Zafar et al., 2023; Homayoonfal, Asemi & Yousefi, 2022). Although each plant exhibits substantial biological activity individually, combining extracts may amplify therapeutic effects through synergistic interactions. Polyherbal formulations may enhance antioxidant capacity, reduce off-target effects, and strengthen cytotoxic actions against cancer cells by integrating complementary phytochemical pathways (Vicol & Duca, 2023; Bouizgma et al., 2023; Jongrungraungchok et al., 2023; Slavova-Kazakova et al., 2021). Plant-derived extracts are increasingly studied not only for clinical therapeutic potential but also as emerging candidates in animal health and veterinary nutrition, where natural antioxidants and immunomodulatory agents may enhance physiological resilience, metabolic balance, and disease resistance. The herbal extract may therefore hold relevance across biomedical and livestock applications. Accordingly, this study evaluates the phytochemical composition, antioxidant activity, and cytotoxic potential of a combined extract of herbal extract, with relevance to both human therapeutic applications and prospective roles in veterinary and nutraceutical research. By integrating biochemical assays, cytotoxic screening, and spectroscopic characterization, the present study aims to provide a foundational assessment of the extract as a multifunctional bioactive agent and to inform future mechanistic, transcriptomic, and *in vivo* investigations.

## Materials & Methods

### Plant materials and extraction procedure

Equal quantities (500 g each) of herbal extract were purchased from local markets in Riyadh, Saudi Arabia. All plant materials were washed, shade-dried, and ground into fine powder. The powders were combined at a 1:1:1 ratio and extracted in distilled water using a hot-water maceration method as described previously (Ammari et al., 2024; Alhimaidi et al., 2024). The extraction procedure was standardized and performed in triplicate across three independent batches to ensure reproducibility. Each batch was processed using identical conditions, and batch consistency was confirmed by comparing total phenolic content and UV–Vis profiles, the pooled extract was filtered, concentrated by rotary evaporation, and stored at 4 °C until analysis.

### Chemicals and reagents

All analytical reagents, solvents, and standards (gallic acid, quercetin, tannic acid, methanol, Folin-Ciocalteu reagent) were purchased from Sigma-Aldrich (St. Louis, MO, USA) and used without further modification.

### Gas chromatography–mass spectrometry (GC-MS) analysis

Chemical constituents were analyzed using a Shimadzu GC-2010 Plus system coupled to a QP2010 Ultra mass spectrometer. A Rt-2560 capillary column (100 m length, 0.25 mm ID, 0.2 µm film) was used with helium as carrier gas (one mL/min). Oven temperature was programmed from 50 °C to 250 °C over 81 min. Mass spectral acquisition ranged from m/z 40–500. Identified phytochemical components were matched against library databases (Table 1).

**Table 1:** GC-MS analysis of aqueous herbal extract.

Peak	Name	Chemical formula	Molecular weight (g/mol)	Rt	Area %
1	1H,6H-Triazolo[4,5-E][1,2,3]-benzotriazole-5-amine	C6H5N7	175.15	13.428	1.411291
2	Phenol, 2,2′-(1,2-ethanediylidenedinitrilo)bis-	C14H12N2O2	240.25	14.838	1.679351
3	2-Oxo-6-phenyl-1,2-dihydro-3,4-pyridinedicarbonitrile	C13H7N3O	221.21	15.101	5.208254
4	[1,1′-Bicyclopropyl]-2-octanoic acid, 2′-hexyl-, methyl ester	C21H38O2	322.52	15.768	7.919701
5	1,3,5-Triazine-2,4-diamine, N,N’-bis(1-methylethyl)-6-(methylsulfonyl)-	C10H19N5O2S	273.35	16.902	1.004724
6	4-Benzyl-1-[4-(4-methoxy-phenyl)-thiazol-2-yl]-piperidine	C22H24N2OS	364.5	23.179	12.02017
7	Acetamide, N-(6-acetylaminobenzothiazol-2-yl)-2-(adamantan-1-yl)-	C21H25N3O2S	383.5	23.309	8.403814
8	Pyridazine-3,5-dicarbonitrile, 1,6-dihydro-4-benzyl-6-oxo-1-phenyl-	C19H12N4O	312.32	32.583	0.834311
9	4-Dehydroxy-N-(4,5-methylenedioxy-2-nitrobenzylidene)tyramine	C16H14N2O4	298.29	34.767	15.50008
10	4-Methyl-6-phenyltetrahydro-1,3-oxazine-2-thione	C11H13NOS	207.29	36.63	1.59811
11	2H-1-Benzopyran, 2,2-diphenyl-	C21H16O	248.35	39.287	1.145746
12	4-(6,7-Dimethoxy-2H-1,3-benzodioxol-5-yl)-1H,4H,6H,8H-pyrazolo[3,4-e][1,4]thiazepin-7-one	C15H15N3O5S	349.36	40.75	4.588507
13	Benzene, 1-(4-diethylaminobenzylidenamino)-4-phenylazo-	C23H24N4	356.46	41.459	0.256646
14	2,3-pyrazinediamine, N2,N3,5,6-tetraphenyl-	C28H22N4	414.5	43.776	20.98104
15	Benzenemethanamine, *α*-[2-imino-2-phenyl-1-(phenylmethyl)ethylidene]-N-phenyl-	C28H24N2	388.5	45.558	2.36099
16	: (+-)-O-Benzylcheilanthifoline	C26H25NO4	415.48	58.414	11.87135
17	Benzenamine, 4,4′-((6-phenyl-2,4-pyrimidinediyl)bis-	C22H18N4	338.4	63.036	2.102658
18	2-[2,2,3,3,3-Pentafluoro-1-(4-phenoxy-phenylamino)-propylidene]-malononitrile	C18H10F5N3O	379.28	73.624	1.113259

### Determination of total phenolic content (TPC)

**Figure 1:**
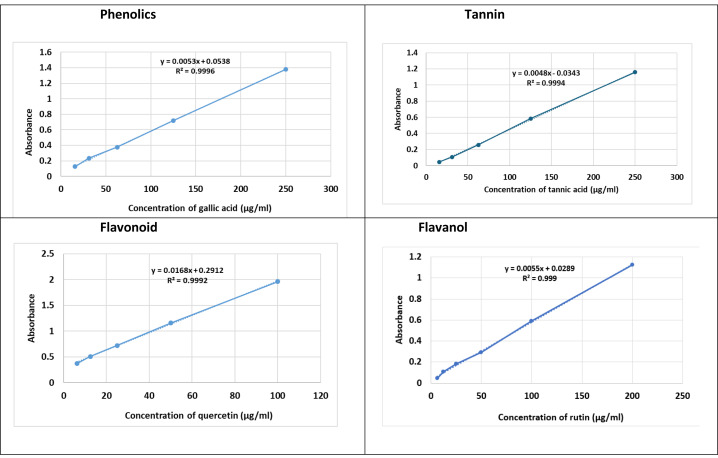
Total of fenugreek TPC, TFC, TF-OL and TTC.

Total phenolic content was quantified using the Folin-Ciocalteu assay. A 100 µL extract sample was mixed with 200 µL Folin reagent (10%) and incubated for 2 h in darkness, followed by addition of 800 µL sodium carbonate (0.7 mM). Absorbance was measured at 765 nm. Results were expressed as mg gallic acid equivalents (GAE)/g dry weight (Table 2) and (Fig. 1).

**Table 2:** Total phenolic (TPC), flavonoid (TFC), flavonol (TF-OL), tannin (TTC), detected in seed extract of herbal. values represent the means ± S.E of three measures.

Extract	GAE/g Phenolics	Tannin GAE/g	Flavonoid mg QE/g	Flavanol mg QE/g
Sidr, Fenugreek, and Black Seed	89.35 ± 0.36	88.60 ± 0.51	28.67 ± 0.09	10.52 ± 0.51

### Determination of total tannin content (TTC)

To quantify tannins, 0.1 mL extract was mixed with 1.5 mL distilled water and one mL Folin-Ciocalteu reagent (Hu et al., 2025), followed by 0.8 mL sodium bicarbonate (pH 7.5). After incubation at 45 °C for 30 min, absorbance was read at 700 nm and expressed as mg tannic acid equivalents (TAE)/g (Table 2) and (Fig. 1).

### DPPH (2,2-diphenyl-1-picrylhydrazyl) radical scavenging assay

The antioxidant activity was assessed using a 0.2 mM 2,2-diphenyl-1-picrylhydrazyl (DPPH) methanolic solution. Equal volumes of extract and DPPH solution were incubated for 30 min at room temperature, and absorbance was measured at 517 nm. IC_5__0_ values were calculated based on inhibition curve plots.

### ABTS (2,2′-azinobis-(3-ethylbenzothiazoline-6-sulfonic acid)) radical cation decolorization assay

ABTS^+^ radicals were generated by mixing seven mM 2,2′-azinobis-(3-ethylbenzothiazoline-6-sulfonic acid) (ABTS) with 2.45 mM potassium persulfate and incubating the reaction mixture in the dark at room temperature for 16 h. The resulting ABTS^+^ solution was diluted with methanol to an absorbance of 0.70 ± 0.02 at 734 nm. Extract samples were incubated with the working ABTS^+^ solution for 7 min, and absorbance was recorded at 734 nm. Trolox served as the standard reference antioxidant for calculating Trolox Equivalent Antioxidant Capacity (TEAC).

### Assay for the scavenging of ABTS radicals

This test measures color loss caused by antioxidants converting ABTS+ radical cations into ABTS and decolorizing them. This protocol assesses antioxidant activity according to Ree et al. ABTS radical cations are made by mixing potassium persulfate (2.45 mM) with ABTS (seven mM) water stock. The operational solution is created by mixing equal volumes of stock solutions, incubation at 25 °C for 16 h without light, and diluting with methanol to achieve an absorbance of 0.70 ± 0.2 units at 734 nm using spectrophotometry. Each experiment used fresh solvent and Trolox as the antioxidant standard, with a calibration curve ranging from 0 to 500 µM. Trolox equivalents (TEAC) levels were measured by combining diluted samples (one mL) with an equal quantity of ABTS+ radical cation solution in test tubes. Absorbance was recorded at 734 nm after 7 min.

### Evaluation of *in vitro* cytotoxicity

#### Cell line

This study employed the human epithelial cell line Caco-2 as an *in vitro* model. Cancer cells were obtained from the American Type Culture Collection (ATCC, Manassas, WV, USA). The cells were cultured in a flask containing complete medium (Invitrogen, Carlsbad, CA, USA), supplemented with 10% fetal bovine serum (FBS) and antibiotics (100 µg/mL streptomycin and 100 U/mL penicillin) at 37 °C in a 5% CO2 atmosphere. The culture media were replaced every 2 to 3 days. Upon reaching 90% confluence, the cells were subjected to sub-culturing.

#### MTT (3-[4,5-dimethylthiazol-2-yl]-2,5 diphenyl tetrazolium bromide) assay

Inhibiting Caco-2 cell growth was the principal test of the raw botanical extract’s bioactivity. The most powerful crude sample and its fractions—hexane, dichloromethane, dichloromethane-ethyl acetate, ethyl acetate-methanol, methanol, and acetic acid—were tested further. The study used 3-[4,5-dimethylthiazol-2-yl]-2,5 diphenyl tetrazolium bromide (MTT) assays at 595 nm with a microplate reader (SunRise, TECAN, Inc., USA) to determine fraction cytotoxicity against Caco-2 cell lines and IC50 values. The growth inhibition experiment used Mosmann’s (1983) method on 96-well plates. Two MTT plates were utilized. We diluted each plant crude extract (50 μL) in 100 μL of DMEM on a dilution plate row by row. 6.3 ng/mL to 1.111 mg/mL. Methanol was the vehicle control, and all treatments were repeated and labeled. In the culture plate, 5 × 104 cells/mL of cell lines were planted in 120 μL 96-well plates and treated with 60 μL of diluted treatments from the dilution plates. After 4 days of CO2 incubation, add 20 μL of MTT salt. Incubating MTT salt for 2 h produced formazan crystals. The solution was carefully aspirated using a vacuum aspirator to preserve formazan crystals. Formazan was dissolved in isopropanol and incubated for 10 min. Plate readers recorded optical densities at 595 nm after 10 min on a shaker. Using Origin 8 software (OriginLab Corporation, Northampton, MA, USA; https://www.originlab.com), the IC50 concentration that inhibited cell growth by 50% was calculated and represented as a percentage of the control value. Each chemical was triple-tested.

### UV–visible spectrophotometry and surface charge (zeta potential)

Zeta potential measurements were performed in triplicate across three independently prepared extract batches. The reported value (−22.1 ± 6.92 mV) represents the mean ± standard deviation, with individual measurements ranging from −15.4 mV to −28.7 mV. All samples were analyzed under identical dilution and pH conditions to ensure consistency.

### Statistical analysis

All analyses were performed in triplicate, and results are reported as mean ± standard deviation (SD). Because the study evaluated a single combined extract rather than multiple experimental groups, inferential statistical tests (*e.g.*, ANOVA, *p*-values) were not applied. Descriptive statistics were used to summarize biochemical and cytotoxicity measurements.

## Results

### Phytochemical composition

The extract contained high levels of phenolic (89.35 ± 0.36 mg GAE/g) and tannin compounds (88.60 ± 0.51 mg GAE/g), with moderate amounts of flavonoids (28.67 ± 0.09 mg QE/g) and flavonols (10.52 ± 0.51 mg QE/g). Since phytochemical contents were quantified from a single combined extract rather than from separate treatment groups, results are reported descriptively. The dominance of phenolic-rich compounds suggests strong antioxidant potential consistent with polyphenol-driven mechanisms (Table 1).

### Antioxidant activity

The extract demonstrated moderate free-radical scavenging capacity, with IC_5__0_ values of 359.93 ± 9.91 μg/mL for DPPH and 292.93 ± 4.24 μg/mL for ABTS assays (Table 3). The lower IC_5__0_ value in ABTS suggests greater affinity toward ABTS^+^ radicals relative to DPPH.

**Table 3:** Inhibition percentage and IC_50_ of herbal extract for DPPH scavenging activities.

Extracts	Antioxidant capacity inhibition%	
	IC_50_ DPPH (μg/mL)	IC_50_ ABTS(μg/ml)
Sidr, Fenugreek, and Black Seed	359.93 ± 9.91	292.93 ± 4.24

### Cytotoxic activity against Caco-2 cells

**Figure 2:**
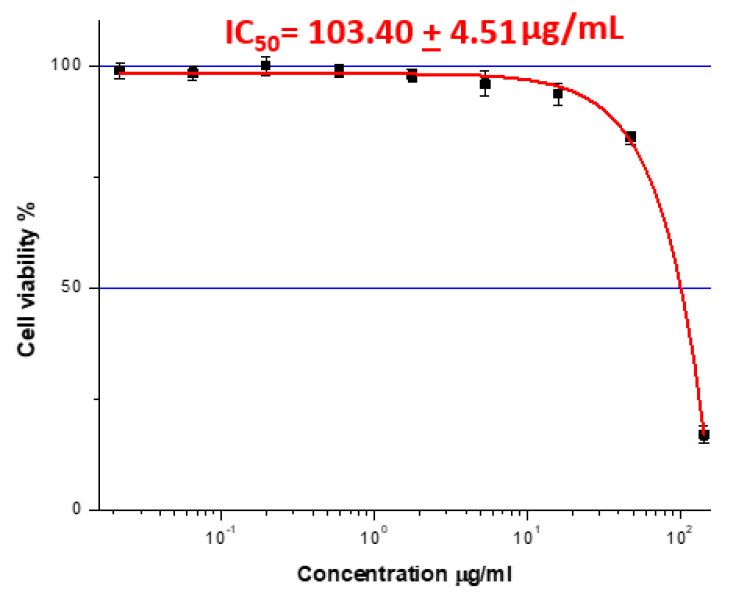
The IC_50_ value represents the plant extract dose that inhibited cancer cell growth by 50% in Caco-2 cells (IC_50_= 103.40 + 4.51 μg/mL.

The extract reduced the viability of Caco-2 colorectal cancer cells in a dose-dependent manner, with an IC_5__0_ value of 103.40 ± 4.51 μg/mL (Fig. 2). Higher concentrations produced noticeably greater inhibition compared to lower doses, confirming the extract’s potential anticancer activity.

### UV–visible spectroscopy and zeta potential

**Figure 3:**
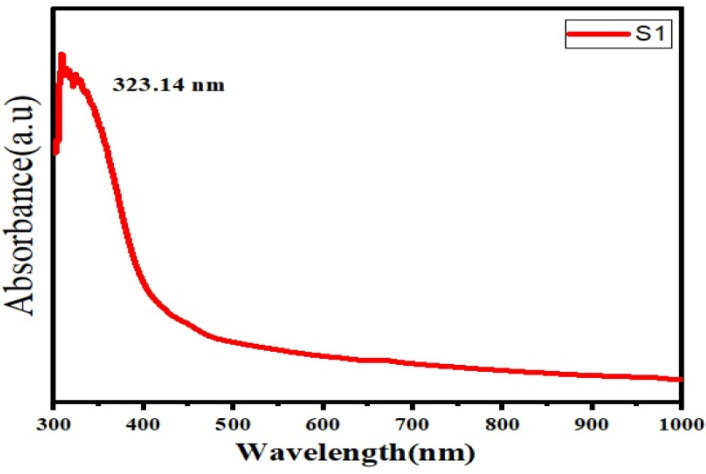
UV spectra of herbal extract.

**Figure 4:**
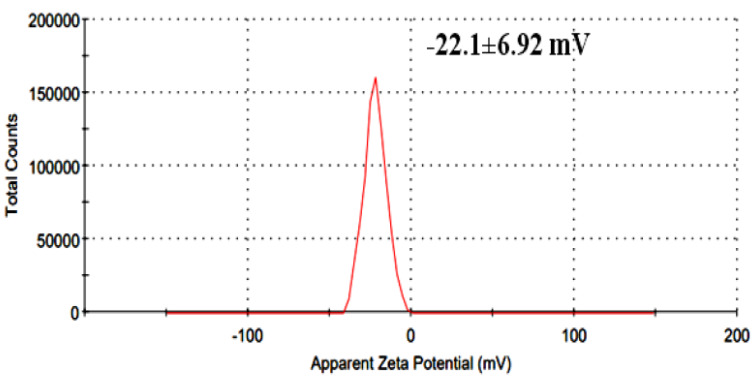
Zeta potential distribution of herbal extract.

UV–Vis analysis showed a major absorption peak at 323.14 nm, confirming the presence of conjugated phenolic and aromatic compounds (Fig. 3). The extract exhibited a mean zeta potential of −22.1 ± 6.92 mV across three independently prepared batches, with values ranging from −15.4 mV to −28.7 mV (Fig. 4). This range indicates moderate colloidal stability, suggesting the extract possesses sufficient electrostatic repulsion to limit aggregation under aqueous conditions. Zeta potential values within −20 to −30 mV is generally associated with dispersions of moderate stability, whereas values exceeding ±30 mV correspond to highly stable nanoparticles. The negative surface charge observed in this extract may influence membrane interactions and facilitate cellular uptake due to enhanced electrostatic binding, thereby contributing to its biological activity and potential therapeutic relevance.

## Discussion

### Antioxidant mechanisms and phenolic contributions

The combined extract of herbal extract demonstrated notable antioxidant properties, consistent with existing research on medicinal plant formulations. The high phenolic and tannin content observed in this study is associated with strong free-radical scavenging activity, a relationship widely reported in plant-derived therapeutics (Chopra & Dhingra, 2021; Chaachouay & Zidane, 2024). Phenolic compounds act as hydrogen donors and electron-transfer agents, reducing oxidative stress that contributes to cancer, metabolic disease, and inflammation (Cragg & Newman, 2013; Thomford et al., 2018).

### Antioxidant activity in radical scavenging assays

The extract exhibited moderate scavenging activity in both DPPH and ABTS assays, comparable to polyherbal mixtures reported to possess synergistic antioxidant properties (Bouizgma et al., 2023; Slavova-Kazakova et al., 2021). The lower IC_5__0_ observed in ABTS may reflect differential interactions with radical species, influenced by the polarity and structural properties of phenolic compounds.

### Anticancer potential and mechanistic evidence

Cytotoxicity against Caco-2 colorectal cancer cells (IC_50_ = 103.40 ± 4.51 μg/mL) suggests anticancer potential, consistent with reports that *Ziziphus*, *Fenugreek*, and *Nigella* species regulate apoptosis and proliferative pathways (Sadeghi, Imenshahidi & Hosseinzadeh, 2023; Sheikhnia et al., 2023; Homayoonfal, Asemi & Yousefi, 2022). Thymoquinone from *Nigella sativa* has been shown to modulate pro-apoptotic signaling and oxidative stress in colorectal cancer cells (Zafar et al., 2023; Khalid et al., 2024), while *Ziziphus* extracts exhibit anti-tumor effects linked to alkaloid and flavonoid constituents (Mongalo, Mashele & Makhafola, 2020; Abdel-Sattar et al., 2024).

Because this study represents an initial bioactivity screening, mechanistic assays such as qPCR or pathway-specific gene profiling were not performed. These analyses are planned for subsequent studies following extract fractionation, consistent with recommendations in pharmacological validation research (Chunarkar-Patil et al., 2024).

### Physicochemical characteristics relevant to formulation

The UV-Vis peak at 323.14 nm confirms the presence of conjugated aromatic phytochemicals, while the zeta potential value (−22.1 ± 6.92 mV) indicates moderate colloidal stability. Such stability may enhance cellular uptake and support future pharmaceutical formulation, consistent with plant-based nanodispersion systems (Padalia & Chanda, 2021).

### Relevance to human therapeutic applications

These findings support evidence from recent polyherbal formulation studies reporting enhanced therapeutic effects through synergistic interactions rather than isolated extracts (Jongrungraungchok et al., 2023; Lahare et al., 2024; Modi et al., 2024). The extract demonstrated potential relevance to human health through mechanisms related to oxidative stress, inflammation, and cancer-associated pathways; however, further mechanistic validation, pharmacokinetic analysis, and clinical evaluation are required before therapeutic applicability can be confirmed.

Potential applications in animal health and nutrition: Beyond human medical relevance, polyphenol-rich herbal extracts are increasingly investigated in animal nutrition and veterinary science, where antioxidant and immunomodulatory effects may support metabolic health, feed efficiency, and oxidative stress resilience. While the current study did not evaluate animal models, future research could explore species-specific dosing and nutraceutical integration.

Although the present study focuses on biochemical and *in vitro* cytotoxicity assays, the biological activities observed are consistent with recent *in vivo* reports demonstrating organ-specific effects of fenugreek extracts. Ammari et al. (2025a) identified hepatic modulation driven by dose-dependent responses in male and female rats, while Ammari et al. (2025b) observed renal protective activity with similar sex-dependent patterns. These animal studies suggest that constituents within the combined extract may influence systemic metabolic and antioxidant pathways *in vivo*, warranting further investigation using whole-organism models.

### Study limitations

This study provides a preliminary biochemical and *in vitro* assessment of a combined extract of Sidr, Fenugreek, and Black Seed. The extract was evaluated as a single polyherbal formulation, and individual plant contributions were not examined separately. Although this approach reflects the intended functional use of the formulation, future studies should include extract fractionation and component-level analyses to determine compound-specific effects.

Additionally, the current work focused on antioxidant and cytotoxic activity using *in vitro* assays. These findings do not establish pharmacokinetic behavior, mechanisms of action, or *in vivo* efficacy. Further research incorporating molecular pathway analysis (*e.g.*, gene expression profiling) and animal models is required to determine therapeutic mechanisms, safety, and bioavailability.

Our results are consistent with recent studies demonstrating antioxidant potential in formulations derived from *Ziziphus* species. For example, Shnawa et al. (2022) reported strong DPPH scavenging activity in nickel oxide nanoparticles synthesized using *Z. spina-christi* extracts, suggesting that phenolic components contribute significantly to redox behavior through metal-complexation–assisted radical neutralization. Similarly, Shnawa et al. (2024) observed notable antioxidant capacity in zinc oxide nanoparticles biosynthesized using *Z. spina-christi* leaf extract, further supporting the role of phytochemicals as mediators of electron-transfer activity. Although the current study does not involve nanoparticle synthesis, the observed IC_5__0_ values for the combined extract fall within the range of antioxidant performance reported for *Ziziphus*-based formulations, indicating that integrating multiple herbal constituents may preserve or enhance such properties through complementary phytochemical interactions.

The antibacterial and anti-inflammatory properties reported for these herbal extracts in previous studies may also contribute to their biological relevance; however, such activities were not evaluated in the present work.

Finally, while spectroscopic and zeta potential analyses provide initial physicochemical characterization, advanced analytical techniques (*e.g.*, LC-MS/MS, HPLC fractionation, and nanoparticle stability assays) would deepen compound identification and formulation stability assessment.

## Conclusions

The combined extract of herbal extract exhibited high phenolic and tannin content and demonstrated notable antioxidant and cytotoxic activity, supporting its potential as a multifunctional natural therapeutic candidate. Future studies will focus on gene-expression profiling and purification of active fractions to elucidate molecular pathways and enhance pharmaceutical applicability.

## Supplemental Information

10.7717/peerj.20824/supp-1 Supplemental Information 1 Gas chromatography–mass spectrometry (GC–MS) profiling data of the herbal extract Identified chemical compounds along with their chemical formulas, molecular weights, retention times (Rt), and relative peak area percentages.

10.7717/peerj.20824/supp-2 Supplemental Information 2 Zeta potential distribution profile of the herbal extract (Sample 1) The surface charge characteristics and stability of the particles as measured in aqueous suspension sample 1.

10.7717/peerj.20824/supp-3 Supplemental Information 3 Zeta potential distribution profile of the herbal extract (Sample 2) The surface charge characteristics and stability of the particles as measured in aqueous suspension sample 2.

10.7717/peerj.20824/supp-4 Supplemental Information 4 Zeta potential distribution profile of the herbal extract (Sample 3) The surface charge characteristics and stability of the particles as measured in aqueous suspension sample 3.

10.7717/peerj.20824/supp-5 Supplemental Information 5 Raw experimental data and calibration curve used for the determination of total phenolic content (TPC) Gallic acid concentrations, corresponding absorbance values, and calculated mean ± standard deviation.

## Abbreviations

*Z. spina-christi*: *Ziziphus spina-christi*
*T. foenum-graecum*: *Trigonella foenum-graecum*
*N. sativa*: *Nigella sativa*
DPPH: 2,2-diphenyl-1-picrylhydrazyl
ABTS: 2,2′-azino-bis(3-ethylbenzothiazoline-6-sulfonic acid)
UV–Vis: Ultraviolet–visible spectroscopy
IC50: Half maximal inhibitory concentration
SD: Standard deviation

## Additional Information and Declarations

### Data Availability

The following information was supplied regarding data availability: The data is available in the Supplemental Files.

## References

[ref-1] Abdel-Sattar M, Al-Obeed RS, Rihan HZ, El-Badan GE (2024). Genetic diversity and relationships among Indian Jujube (*Ziziphus mauritiana* Lamk.) cultivars using morphometric characteristics, matK barcoding, and ISSR markers. Diversity.

[ref-2] Alhimaidi AR, Ammari AA, Amran RA, Rady AM (2024). Assessment of biochemical and histological effects of one of *Ziziphus* genus extract on ovarian function in female rats. Indian Journal of Animal Research.

[ref-3] Ammari AA, Alhimaidi AR, Amran RA, Aljawdah H, Murshed M, Rady AM (2025b). Fenugreek’s nephroprotective potential: sex-specific and dose-dependent effects on kidney function in healthy rats. Arquivo Brasileiro de Medicina Veterinária E Zootecnia.

[ref-4] Ammari AA, Alhimaidi AR, Amran RA, Aljawdah H, Rady AM (2025a). Investigating the dose- and sex-specific impacts of fenugreek (*Trigonella foenum-graecum*) extract on liver enzymes and histology in male and female rats. Arquivo Brasileiro de Medicina Veterinária E Zootecnia.

[ref-5] Ammari AA, Alhimaidi AR, Amran RA, Rady AM (2024). The possible side effects of *Ziziphus spina-christi* extract on the liver, kidneys of female rats. Indian Journal of Animal Research.

[ref-6] Banday AH, ul Azha N, Farooq R, Sheikh SA, Ganie MA, Parray MN, Mushtaq H, Hameed I, Lone MA (2024). Exploring the potential of marine natural products in drug development: a comprehensive review. Phytochemistry Letters.

[ref-7] Bouizgma K, Abourriche A, Rabbah N, Zakari A (2023). Food strategy: antioxidants synergistic effect of natural plant extracts. Journal of Analytical Sciences and Applied Biotechnology.

[ref-8] Chaachouay N, Zidane L (2024). Plant-derived natural products: a source for drug discovery and development. Drugs and Drug CandIdates.

[ref-9] Chopra B, Dhingra AK (2021). Natural products: a lead for drug discovery and development. Phytotherapy Research.

[ref-10] Chunarkar-Patil P, Kaleem M, Mishra R, Ray S, Ahmad A, Verma D, Bhayye S, Dubey R, Singh HN, Kumar S (2024). Anticancer drug discovery based on natural products: from computational approaches to clinical studies. Biomedicines.

[ref-11] Cragg GM, Newman DJ (2013). Natural products: a continuing source of novel drug leads. Biochimica Et Biophysica Acta (BBA)—General Subjects.

[ref-12] Egigu M, Mogesse S (2024). Ethnobotanical study of medicinal plants used to treat human ailments in Oda-Bultum district, west Hararghe zone of Oromia region, Ethiopia. Ethnobotany Research and Applications.

[ref-13] Homayoonfal M, Asemi Z, Yousefi B (2022). Potential anticancer properties and mechanisms of thymoquinone in osteosarcoma and bone metastasis. Cellular & Molecular Biology Letters.

[ref-14] Hu Z, Xiong W, Liang Q, Wang C, Xuan P, Li Y, Hua X, Guo H, Yao Y (2025). Canolol as a key equivalent for phenolic content quantification in rapeseed oil *via* Folin–Ciocalteu method. European Food Research and Technology.

[ref-15] Jongrungraungchok S, Madaka F, Wunnakup T, Sudsai T, Pongphaew C, Songsak T, Pradubyat N (2023). *In vitro* antioxidant, anti-inflammatory, and anticancer activities of mixture Thai medicinal plants. BMC Complementary Medicine and Therapies.

[ref-16] Kalantari E, Pournamdari M, Mehrabani M, Tarzic ME, Mohamadi N, Asadi A, Sheida I, Sharififar F (2024). Investigation of bioactive fractions from fenugreek seeds (*Trigonella foenum-graecum* L.): chemical profiling, *in vitro* anti-inflammatory activity, and their potential against LPS-stimulated J774A. 1 cells. Advances in Traditional Medicine.

[ref-17] Khalid JP, Martin TM, Prathap L, Nisargandha MA, Boopathy N, Kumar MSK (2024). Exploring tumor-promoting qualities of cancer-associated fibroblasts and innovative drug discovery strategies with emphasis on thymoquinone. Cureus.

[ref-18] Khatoon N, Savita KK, Chaudhary JS, Chanchal DK, Son M (2024). Fenugreek use: biological role of fenugreek in health of human being—a review. Journal of Advanced Zoology.

[ref-19] Kumar N, Ahmad AH, Singh SP, Pant D, Prasad A, Rastogi SK (2021). Phytochemical analysis and antioxidant activity of *Trigonella foenum-graecum* seeds. Journal of Pharmacognosy and Phytochemistry.

[ref-20] Lahare SH, Lahare KH, Lonsane JR, Girbane Y, Chouthe E (2024). Formulation and evaluation of herbal ointment containing Neem and Turmeric extract. World Journal of Pharmaceutical Science and Research.

[ref-21] Modi J, Rathore S, Dwivedi S, Saraogi G (2024). Formulation and evaluation of multipurpose herbal cream. International Journal of Newgen Research in Pharmacy & Healthcare.

[ref-22] Mongalo NI, Mashele SS, Makhafola TJ (2020). Ziziphus mucronata Willd. (Rhamnaceae): it’s botany, toxicity, phytochemistry and pharmacological activities. Heliyon.

[ref-23] Mosmann T (1983). Rapid colorimetric assay for cellular growth and survival: application to proliferation and cytotoxicity assays. Journal of Immunological Methods.

[ref-24] Oubannin S, Jadouali SM, Atifi H, Bijla L, Ibourki M, Gagour J, Ait Bouzid H, Ait Aabd N, Bouyahya A, Harhar H, Goh KW, Ming LC, Razi P, Gharby S (2024). Antioxidant activity, physico-chemical properties, and bioactive compounds of *Nigella sativa* seeds and oil impacted by microwave processing technique. Heliyon.

[ref-25] Padalia H, Chanda S (2021). Synthesis of silver nanoparticles using *Ziziphus nummularia* leaf extract and evaluation of their antimicrobial, antioxidant, cytotoxic and genotoxic potential (4-in-1 system). Artificial Cells, Nanomedicine, and Biotechnology.

[ref-26] Pourahmadi M, Fathi M, Rahimipour M, Shaterian N, Jahromi HK (2023). Hydroalcoholic extract of *Ziziphus jujuba* leaf to prevent ethylene glycol and ammonium chloride-induced kidney stones in male rat: is it effective?. Urology Journal.

[ref-27] Sadeghi E, Imenshahidi M, Hosseinzadeh H (2023). Molecular mechanisms and signaling pathways of black cumin (*Nigella sativa*) and its active constituent, thymoquinone: a review. Molecular Biology Reports.

[ref-28] Shadab M, Akhtar N, Siddiqui MB (2024). Medicinal and nutritional importance of *Trigonella foenum-graecum* in human health. Medicinal plants and their bioactive compounds in human health: volume 1.

[ref-29] Sheikhnia F, Rashidi V, Maghsoudi H, Majidinia M (2023). Potential anticancer properties and mechanisms of thymoquinone in colorectal cancer. Cancer Cell International.

[ref-30] Shnawa BH, Jalil PJ, Al-Ezzi A, Mhamedsharif RM, Mohammed DA, Biro DM, Ahmed MH (2024). Evaluation of antimicrobial and antioxidant activity of zinc oxide nanoparticles biosynthesized with *Ziziphus spina-christi* leaf extracts. Journal of Environmental Science and Health, Part C.

[ref-31] Shnawa BH, Jalil PJ, Hamad SM, Ahmed MH (2022). Antioxidant, protoscolicidal, hemocompatibility, and antibacterial activity of nickel oxide nanoparticles synthesized by *Ziziphus spina-christi*. Bionanoscience.

[ref-32] Singh S, Chaurasia PK, Bharati SL (2023). Hypoglycemic and hypocholesterolemic properties of Fenugreek: a comprehensive assessment. Applied Food Research.

[ref-33] Slavova-Kazakova A, Janiak MA, Sulewska K, Kancheva VD, Karamać M (2021). Synergistic, additive, and antagonistic antioxidant effects in the mixtures of curcumin with (−)-epicatechin and with a green tea fraction containing (−)-epicatechin. Food Chemistry.

[ref-34] Thomford NE, Senthebane DA, Rowe A, Munro D, Seele P, Maroyi A, Dzobo K (2018). Natural products for drug discovery in the 21st century: innovations for novel drug discovery. International Journal of Molecular Sciences.

[ref-35] Vicol C, Duca G (2023). Synergistic, additive, antagonistic effects and the prooxidant character of antioxidants: interactions in natural compounds. Fundamental and biomedical aspects of redox processes.

[ref-36] Zafar I, Safder A, Imran Afridi H, Riaz S, ur Rehman R, Unar A, Nisa FU, Gaafar A-Z, Bourhia M, Wondmie GF, Sharma R, Kumar D (2023). *In silico* and *in vitro* study of bioactive compounds of *Nigella sativa* for targeting neuropilins in breast cancer. Frontiers in Chemistry.

