# Supplementary figures and images for: In vitro bioactivity and phytochemical characterization of a polyherbal extract with antioxidant and anticancer properties
Source: PeerJ. 2026 Feb 17;14:e20824. doi: 10.7717/peerj.20824 (PMC12922583; doi:10.7717/peerj.20824)

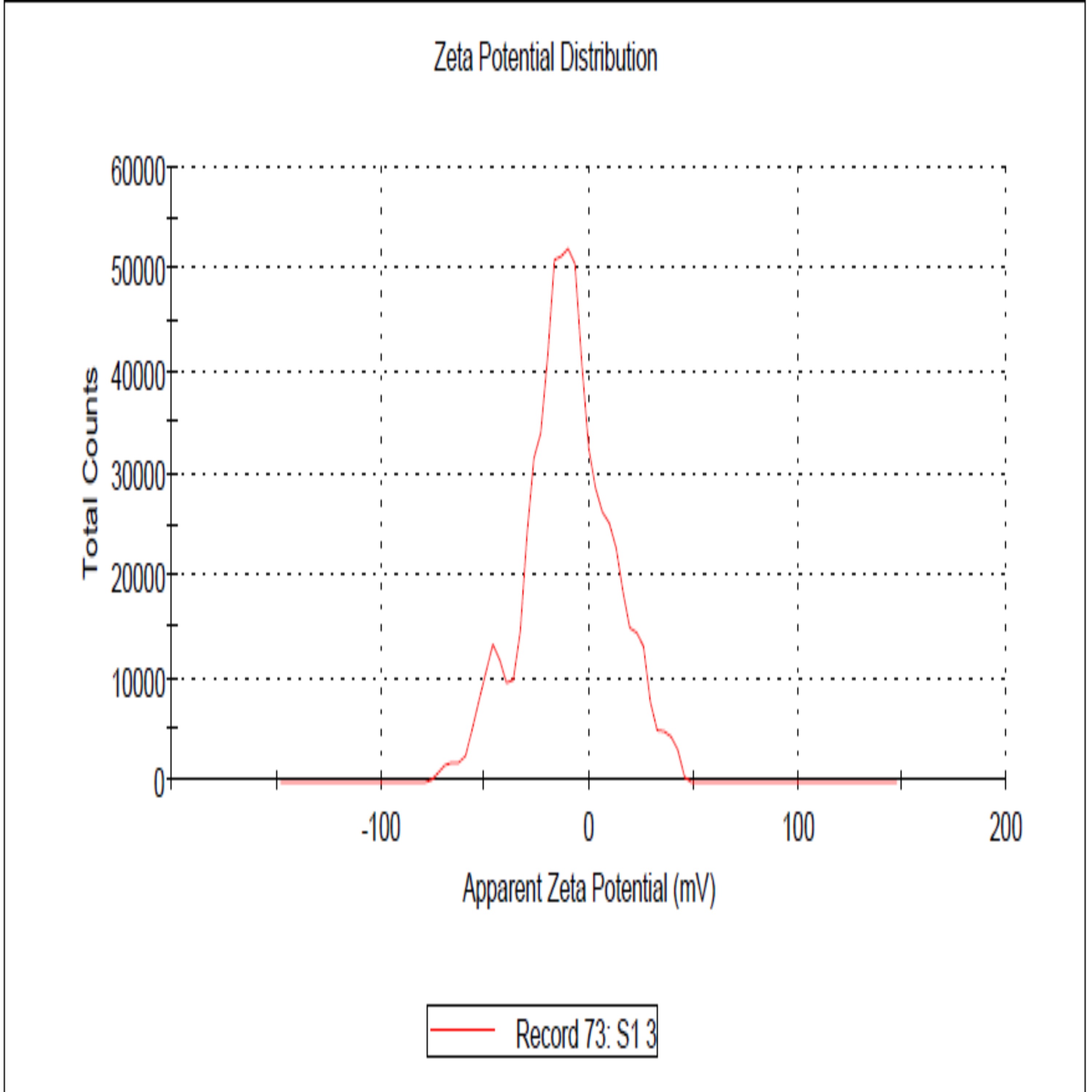

Supplement: Supplemental Information 2 — The surface charge characteristics and stability of the particles as measured in aqueous suspension sample 1. [file peerj-14-20824-s002.jpg]

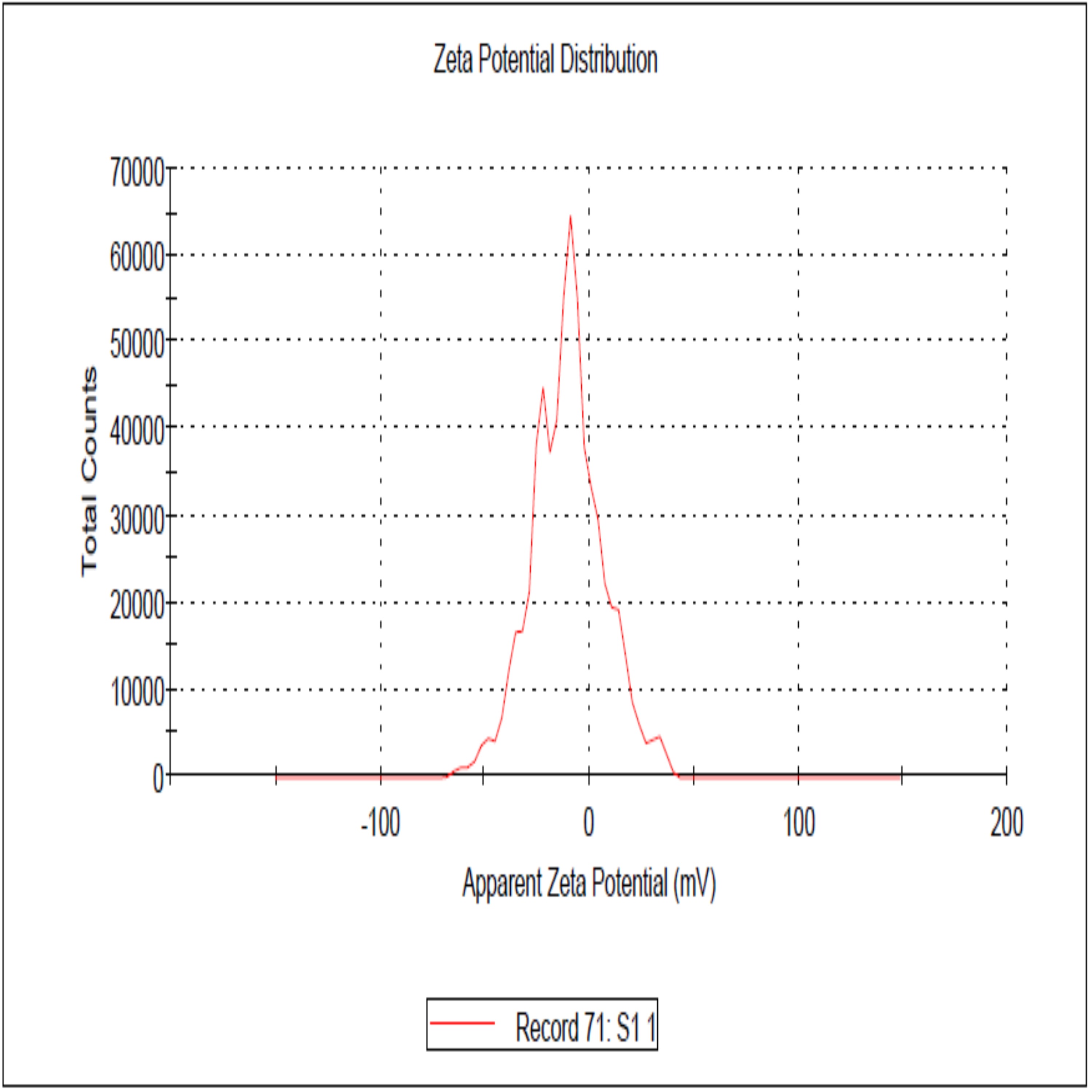

Supplement: Supplemental Information 3 — The surface charge characteristics and stability of the particles as measured in aqueous suspension sample 2. [file peerj-14-20824-s003.jpg]

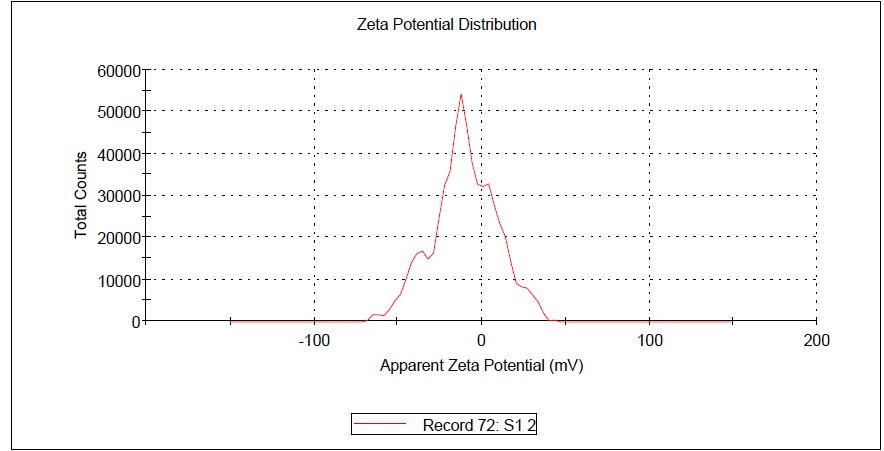

Supplement: Supplemental Information 4 — The surface charge characteristics and stability of the particles as measured in aqueous suspension sample 3. [file peerj-14-20824-s004.png]
